# Great-grandparenthood as a late-life family role: identity and continuity

**DOI:** 10.1093/geront/gnag084

**Published:** 2026-05-04

**Authors:** Zuzana Talašová

**Affiliations:** Department of Sociology, Faculty of Social Studies, Masaryk University, Brno, Czech Republic

**Keywords:** Intergenerational relationships, Narrative gerontology, Life course perspective, Kinship roles, Relational aging

## Abstract

**Background and Objectives:**

This article examines great-grandparenthood as a late-life family role that has remained overlooked in gerontological and sociological research. The study draws on life course theory, critical and narrative gerontology, and the concept of doing family to explore how great-grandparents perceive and negotiate this role in later life.

**Research Design and Methods:**

Based on qualitative, timeline-based interviews with ten participants, the analysis applies a thematic narrative approach to examine how meanings of intergenerational connection, family identity, and aging are constructed across the life course.

**Results:**

Findings suggest that great-grandparenthood is rarely recognized as a distinct identity and tends to remain relationally and culturally invisible. The role does not emerge automatically from biological status but is enacted through episodic relational engagement, such as presence, emotional rituals, and shared time. It is also shaped by symbolic recognition within family narratives and generational positioning. Positioned at the margins of family life, great-grandparenthood emerges as a liminal late-life identity reflecting broader dynamics of aging, intergenerational relations, and cultural scripts.

**Discussion and Implications:**

These findings contribute to gerontological debates on family roles and relational aging by highlighting the symbolic and narrative dimensions of very late-life family positions. They also point to the need for future research incorporating intergenerational perspectives and comparative cultural contexts.

Great-grandparenthood has long existed as a familial role, yet it has only recently emerged as a sociologically relevant category in light of population aging and extended generational structures. Although demographic changes have increased the presence of great-grandparents in contemporary families (Troll, 1971), their role remains largely overlooked in sociological and anthropological research. Existing studies often reduce great-grandparenthood to symbolic presence or a marker of generational continuity (Doka & Mertz, 1988; Even-Zohar & (Tzurit) Garby, 2016; Roberto & Skoglund, 1996; Wentowski, 1985), with limited attention to how individuals themselves perceive and negotiate this role across the life course. Where great-grandparenthood is addressed more directly, studies tend to privilege emotional meanings and formal descriptions over relational practices and everyday negotiation (Castañeda-García et al., 2017; Dias & Pinto, 2007). This article fills this gap by exploring great-grandparenthood as a late-life family role shaped by biography, intergenerational dynamics, and cultural expectations. To capture how this role is enacted and interpreted within family life, this study draws on Morgan’s (2011) concept of *doing family* and his notion of personal life. The study approaches great-grandparenthood as both a relational and symbolic family role. The relational aspect highlights how intergenerational ties are created and sustained through everyday practices of care, recognition, and shared time (Finch, 2007; Brannen, 2006). The symbolic aspect, in turn, captures how meanings of family roles reflect broader moral and cultural expectations (Gubrium & Holstein, 1990; Smart, 2011). Drawing on life course theory and critical gerontology, it emphasizes the symbolic, relational, and narrative aspects of the role. The guiding question is: *How do great-grandparents perceive their role in later life, and how do they negotiate it across generations?*

Great-grandparenthood is approached as a relational family role that does not emerge automatically from biological status but is enacted through everyday family practices and forms of recognition. Drawing on Morgan’s (2011) concept of doing family, family roles are understood as produced through interaction, shared time, and moral expectations rather than fixed positions (Finch, 2007; Morgan, 2011). The analysis also attends to symbolic presence, referring to the ways great-grandparents remain part of family life through emotional, narrative, or moral significance even in the absence of regular caregiving (Gubrium & Holstein, 1990; Smart, 2011). Finally, the concept of relational invisibility is introduced to capture situations in which great-grandparenthood exists biologically but lacks social recognition, narrative articulation, or a clearly defined place within family relations.

Focusing on great-grandparenthood through symbolic, relational, and narrative lenses allows for a comprehensive understanding of how late-life family roles are produced, maintained, and reinterpreted. The symbolic dimension foregrounds how family roles embody broader moral and cultural meanings. For example, being a “great-grandparent” may signify continuity, lineage, or moral authority within the kin network (Gubrium & Holstein, 1990; Smart, 2011). The *relational* dimension emphasizes the performative and interactional nature of kinship, drawing on Morgan’s (2011) concept of *doing family* to reveal how intergenerational bonds are enacted and negotiated through everyday practices of care, time, and recognition. The *narrative* dimension, informed by critical and narrative gerontology (Baars, 1991; Gilleard & Higgs, 2010), highlights how individuals make sense of aging and kinship through storytelling, thus linking biography with cultural expectations. Bringing these three perspectives together provides an interpretive framework that captures both the structural and experiential facets of family life. It enables an analysis of great-grandparenthood as a site where personal histories intersect with social norms and where aging becomes a relational process of meaning-making.

## Literature review

### Family roles across the life course

Role transitions—such as becoming a parent or great-grandparent—are shaped by biography, relationships, and cultural expectations. These transitions are not automatic but negotiated through interaction, care, and recognition. Demographic changes like increased longevity and delayed parenthood have led to more “verticalized” families (Bengtson, 2001), creating new intergenerational complexities (Chisholm, 1999; Fine & Glendinning, 2005; Hochschild, 2012). While great-grandparents are often described as carriers of continuity or family wisdom (Doka & Mertz, 1988; Roberto & Skoglund, 1996; Wentowski, 1985), few studies examine how these meanings are enacted or disrupted in practice (Even-Zohar & (Tzurit) Garby, 2016; Gibson & Houser, 2007; Gordon, 2018; Pacheco Barzallo et al., 2024).

According to life course theory, roles are embedded in broader social structures—including norms, class, and kinship patterns—that shape how responsibilities and identities are transferred and reinterpreted (Crosnoe et al., 2004; Macmillan & Copher, 2005; Sewell, Jr., 1992). Following Elder’s (1998) principle of linked lives, this study views great-grandparenthood as shaped by both individual biographies and interconnected generational trajectories, sensitive to timing and societal change (Goldin, 2014; Kan et al., 2011).

### Post-socialist context and cultural continuities

The Czech context offers a particularly insightful setting for studying great-grandparenthood and intergenerational relations. As a post-socialist society, the Czech Republic combines long-standing norms of intergenerational solidarity with significant transformations in gender, care, and family life since the 1990s (Hájek & Vann, 2015; Souralová, 2018). The legacy of state socialism, which combined full female employment with persistent domestic caregiving expectations (Hájek & Vann, 2015; Souralová, 2018; True, 2003), continues to influence family roles, particularly in late life (Ferber & Raabe, 2003; Saxonberg, 2013). These historical and institutional continuities make it a meaningful case for examining how late-life family roles are experienced and negotiated within changing expectations of kinship and care. While this study does not focus explicitly on gender, such cultural scripts shape how great-grandparenthood is perceived and enacted in everyday contexts.

### Theoretical and conceptual framework

This study draws on four complementary theoretical perspectives. First, the concept of doing family (Morgan, 2011) conceptualizes great-grandparenthood as a relational practice enacted through everyday interactions rather than fixed positions. Second, a symbolic perspective on family roles (Gubrium & Holstein, 1990; Smart, 2011) highlights how moral meanings and cultural expectations shape kinship roles. Third, life course theory (Elder, 1998) provides a temporal lens, emphasizing linked lives, transitions, and historical context. Fourth, insights from critical and narrative gerontology (Baars, 1991; Gilleard & Higgs, 2010) foreground reflexivity, storytelling, and the construction of late-life identities.

While previous studies have often addressed great-grandparenthood primarily in symbolic or descriptive terms, this framework integrates relational, symbolic, and temporal perspectives to conceptualize the role as culturally situated and dynamic. The concept of doing family (Morgan, 2011) serves here not as a descriptive label but as an analytical lens for examining how great-grandparenthood is enacted in everyday interactions and interpreted across generations. At the same time, the symbolic dimension of family life (Gubrium & Holstein, 1990; Smart, 2011) highlights how meanings and moral values attached to kin roles are shaped by cultural expectations. It also draws attention to shared cultural understandings and assumptions about kinship. Together, these perspectives frame great-grandparenthood as both a lived relationship and a cultural construct that unfolds across time. Life course theory (Elder, 1998) provides a temporal lens for linked lives and role transitions, while critical and narrative gerontology (Baars, 1991; Gilleard & Higgs, 2010; Katz, 1996; van Dyk, 2014; Waddell et al., 2024) foregrounds identity work through reflection and storytelling and connects family roles to questions of meaning in later life. This perspective links relational and symbolic dimensions of family life to questions of meaning and aging. Recent studies also emphasize the importance of intergenerational relationships and family support for older adults’ well-being and identity (Silverstein et al., 2025). Finally, insights from post-socialist family studies (Connell, 2002; Hájek & Vann, 2015) provide contextual insight into how historical legacies and institutional continuities shape intergenerational roles and family expectations in the Czech Republic.

## Methodology

### Analytical orientation

This study frames great-grandparenthood as a role actively constructed through relational practice and biographical reflection. Drawing on doing family, it treats the role as enacted across time and generations—meaningful where there is contact and recognition, and fragile or liminal where these are absent. The concept of relational invisibility is introduced to capture cases where the biological role exists but lacks performative enactment or symbolic validation. This lens reveals how late-life family roles may become muted or marginalized in the absence of recognition and intergenerational engagement.

### Research design and data collection

A qualitative, interpretive methodology was selected to capture the relational and experiential dimensions of late-life family roles. Ten semi-structured interviews with great-grandparents were conducted using purposive sampling. Great-grandparents were purposively selected as information-rich cases occupying a unique late-life position, not for representativeness but for insight into how intergenerational roles are experienced and negotiated. Participants were recruited through local community centers and family networks, and interviews were conducted in their homes to ensure comfort and a natural conversational setting. Inclusion criteria included the ability to reflect on family experiences and willingness to share intergenerational stories. Each interview was conceived as a co-constructed and reflexive encounter (Holstein & Gubrium, 2003), in which meaning emerged in dialogue between participant and researcher. Interviews covered domains such as intergenerational relationships, caregiving experiences, role transitions, and reflections on family continuity. To facilitate temporal reflection and support participants’ meaning-making, each participant engaged in a timeline-based interview (Adriansen, 2012; Bagnoli, 2009; Basnet et al., 2023; Berends, 2011). The use of visual timelines draws on qualitative and life-course methodologies that integrate narrative and visual elicitation techniques to support memory recall and biographical reflection (Adriansen, 2012; Sheridan et al., 2011; Kolar et al., 2015).

During the interviews, participants co-constructed visual representations of their life trajectories, identifying key family milestones, caregiving episodes, and intergenerational transitions. They generated the events independently and reflected on each life stage, providing insight into how they constructed and negotiated meanings around their evolving family roles. This process created a flexible narrative structure guided by participants themselves, linking biographical memories with intergenerational experiences. The interviews were audio-recorded, transcribed verbatim, and conducted between autumn 2023 and autumn 2024. While participants varied in socio-economic background, this analysis focused on intergenerational roles across cases rather than comparative social positioning. Table 1 summarizes the characteristics and narrative foci of the ten interviewed great-grandparents. The thematic focus was derived through inductive analysis of interviews and visual timelines, identifying recurring motifs such as caregiving, withdrawal, religious transmission, and emotional continuity. While each narrative is unique, the table highlights shared concerns around role recognition, symbolic participation, and intergenerational connection.

**Table 1:** Characteristics and thematic focus of participants.

Pseudonym	Gender	Age	Narrative focus
**Marie**	Female	76	Tension over upbringing, lack of recognition of past care
**Petr**	Male	84	Attachment to family space, disappearance of role
**Libuše**	Female	86	Emotional support, symbolic resistance to labeling
**Božena**	Female	91	Embodied withdrawal from active role
**Růžena**	Female	79	Desire for continued presence, symbolic
**Daniela**	Female	82	Supportive role, no active transmission
**Vladimír**	Male	88	Focus on survival, not on new roles
**Eliška**	Female	80	Emphasis on traditional gender roles
**Oldřich**	Male	78	Uncertainty about position in family
**Anna**	Female	87	Faith and religious transmission are ignored

*Note.* Data from the author’s fieldwork (2025).

### Data analysis

The combined dataset—interview transcripts and visual timelines—was analyzed using Atlas.ti software (ATLAS.ti Scientific Software Development GmbH, Berlin, Germany). An inductive, theory-informed approach guided the analysis, drawing from life course theory, narrative gerontology, and the concept of doing family.

Open coding was first applied to interview transcripts to identify initial categories such as caregiving, relational positioning, autonomy, continuity, and legacy. These were then grouped into higher-level themes reflecting temporal patterns, relational shifts, and key turning points in family life.

The visual timelines were analyzed in five steps adapted from Basnet et al. (2023): (1) familiarization with each timeline; (2) identification of temporal themes such as caregiving and reciprocity; (3) cross-case comparison; (4) identification of turning points; and (5) integration with narrative data. This process traced the evolving meaning of great-grandparenthood across biographical trajectories.

The analysis was conducted solely by the author, who maintained analytic rigor through iterative reading, reflexive memoing, and ongoing comparison between cases and data sources. Field notes and contextual observations were used to support interpretation but were not formally coded.

### Reflexivity

As a younger researcher sharing generational proximity with participants’ grandchildren, attention was given to how this positioning shaped the interview setting and the narratives produced. While this proximity likely facilitated openness and trust, it also introduced asymmetries of age and experience that required sustained reflexive awareness. Interviews were approached as co-constructed sites of narrative production (Holstein & Gubrium, 2003), with careful attention to participants’ agency in defining their roles and experiences. Rather than treating accounts as transparent representations, consideration was given to how the researcher’s generational standpoint might subtly influence what was emphasized, softened, or left unsaid.

### Ethical considerations

All research procedures followed institutional ethical standards. This study was deemed exempt from ethics board review by *Anonymized for Review*. Care was taken with respect to participants’ advanced age and potentially sensitive topics related to family ties and personal histories. Informed consent was obtained, and participants were informed about the study’s purpose, data handling procedures, their rights, and the voluntary nature of their participation. Pseudonyms were used in the transcripts, and identifying details were removed. Interviews were conducted sensitively, with attention to age-related vulnerability and emotional well-being. Data were stored securely and accessed only by the researcher.

## Results

The following three-part analysis presents patterns observed in four-generation families. Section I describes temporality and narrative based on participants’ visual timelines. Section II reports practices related to everyday activities, space, and bodily presence. Section III summarizes cross-cutting patterns to be further discussed in the Discussion.

### Section I: Temporality, narrative, and the visual structure of family identity

**Figure 1:**
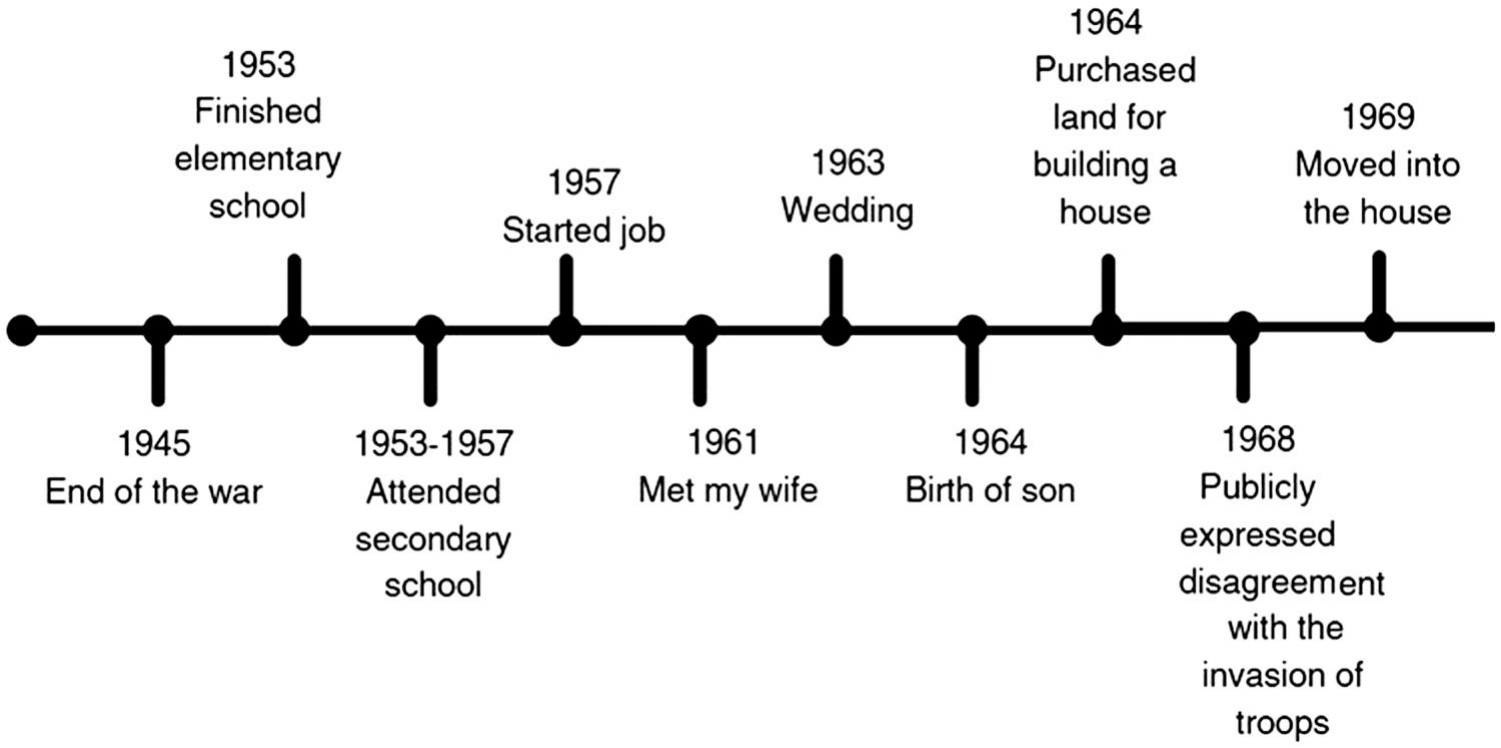
Segment of a male participant’s timeline showing selected events from early to midlife. *Note*. Data from the author’s fieldwork (2025).

**Figure 2:**
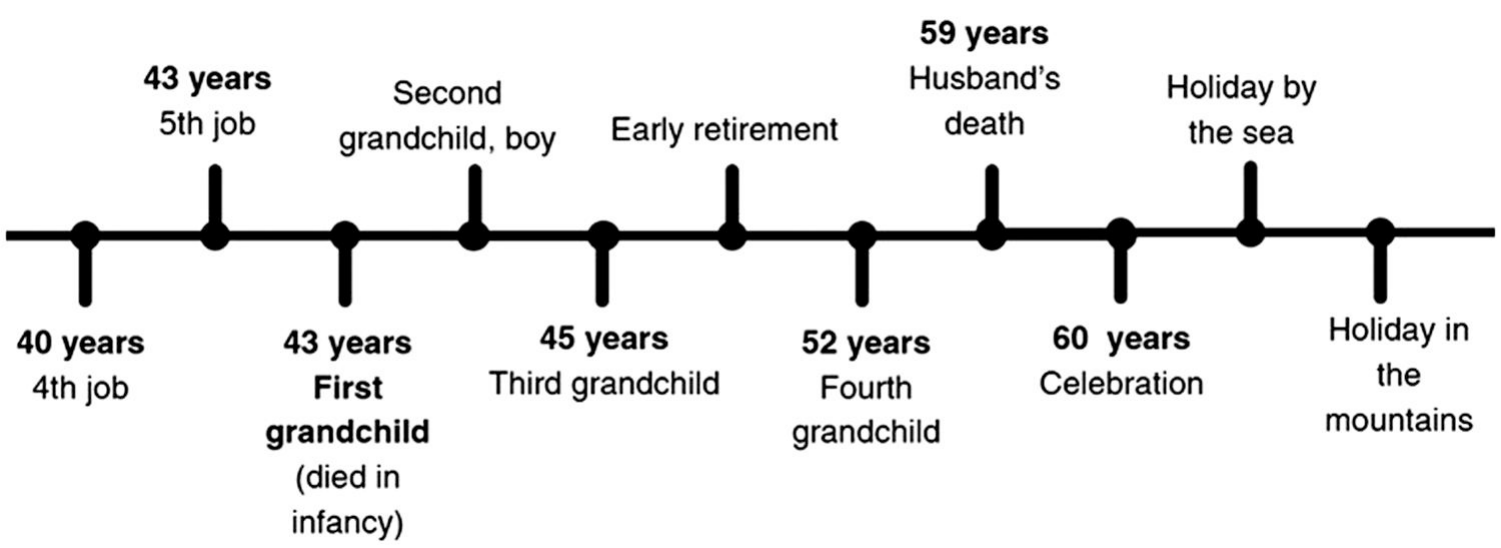
Final segment of a female participant’s timeline showing events from midlife into old age. *Note*. Data from the author’s fieldwork (2025).

The visual timelines created by participants recorded biographical milestones and the presence or absence of references to family roles. Mentions of great-grandparenthood, when present, were typically brief and implicit. The timelines also showed how events were placed across the life course, indicating when family-related entries increased, clustered, or declined. Figure 1 below offers an anonymized example of such a timeline, capturing key life events and relational transitions across several decades. This visual narrative illustrates how care, place, work, and public engagement intersect in personal histories, while also showing the absence of later-life family roles such as great-grandparenthood. Figure 2 provides a contrasting example from a female participant, depicting the later stages of life and the selective recording of family and biographical events.

Across participants’ timelines, recurring motifs emerged, reflecting distinct life trajectories. Five main thematic lines were identified: (a) care and reproduction, (b) work and public engagement, (c) experience of loss, (d) spatial rootedness, and (e) generational layering.

#### Care and reproductive trajectories

Women’s timelines are structured around caregiving and reproductive milestones—marriages, childbirth, childrearing, and the entrance of new generations into family life. Although the word "care" often does not appear explicitly, its presence is reflected in the choice of events and their emotional resonance. The timeline becomes a form of symbolic work—a confirmation of identity as mother, grandmother, or great-grandmother, aligned with service and responsibility for family continuity.

##### Work and spatial anchoring

Male participants emphasized professional and spatial dimensions—key jobs, agricultural work, house construction, or the decision to remain in a home village. Family was mentioned peripherally, mostly in support of public roles. Identity was enacted through work, stability, and spatial anchoring—through meaningful places and activities rather than relational ties.

References to places—villages, ancestral homes, gardens—served as temporal-spatial anchors that linked generations to specific locations and expressed duration, belonging, and rootedness. The home, as a site of transmission or return, often represented the final point where family was done—through visits, repeated rituals, or shared history.

##### Loss and biographical turning points

Key events such as the death of a partner or child were not just recorded but functioned as narrative ruptures—points where time breaks, rhythm shifts, and identity transforms. These moments marked life transitions and often changed the ability to do family—through solitude, changes in daily routines, or loss of contact with younger generations. Certain events stood out on the timelines as clear turning points that interrupted narrative continuity or marked a role transition. Most commonly, the death of a spouse functioned as such a point, often followed by a decline in further event recording.

Another common turning point was the birth of a first child—present across all timelines. In some cases, career changes—joining a collective farm, retirement, or a job shift—also marked turning points, redefining daily life and family positions.

Across cases, the main turning points were related to family events such as marriage and childbirth, and to life changes such as bereavement or retirement. Becoming a great-grandparent, however, was not recorded as a distinct life event, even when it could be inferred from the data. Turning points were concentrated mainly in early and middle adulthood, while later life contained fewer recorded transitions.

##### Generational layering and role shifts

Each timeline revealed a sense of generational layering—marked by events such as the birth of a new generation, children’s weddings, or the arrival of grandchildren. These stages were not always explicitly labelled, but conveyed a visual rhythm of succession, shifting from active caregiving toward more observational roles. While some timelines acknowledged the arrival of new generations, these moments did not tend to reshape the participant’s generational self-positioning in any explicit way.

**Late-life sparsity and role ambiguity.** Comparison of ten cases revealed distinct ways in which respondents constructed their biographies through time and relationships. While women often emphasized care, sacrifice, and family continuity, men framed their identity through professional histories, place, and values. Women’s timelines were more detailed in terms of family events, while men’s emphasized stability and public roles. A noteworthy pattern was the differing density of narrative—middle age was often richly documented, while later life appeared empty. From the perspective of doing family, this suggests that with age, opportunities, and recognition to actively do family decrease. The role of great-grandparent was not explicitly identified in any case, indicating its cultural ambiguity and relational absence.

The timelines showed that participants selected events according to what they considered important, rather than aiming for a complete record. The visual timelines emphasized relational aspects of family life, including moments of emotional connection, shared responsibility, and silence. Gendered patterns were clear: women focused on caregiving and kinship ties, men on work and place. Yet across both, the later stages of life were sparsely represented and largely unmarked by explicit roles. Mentions of great-grandchildren, when they appeared, were typically indirect and lacked narrative elaboration. The role itself hovered on the margins of the family story—acknowledged but seldom explored or given expressive weight. Great-grandparenthood appeared as a rarely elaborated aspect of late-life family experience. While Section I highlights how great-grandparenthood appears—or remains absent—in narrative and visual structure, the following section turns to everyday practices through which this role is enacted in later life.

### Section II: Doing family in later life: great-grandparenting through practices, space, and the body

This section draws explicitly on participants’ interview accounts to examine how great-grandparents describe and enact their role in everyday life. In these accounts, great-grandparents described participating in family life in subtle, symbolic, or irregular ways that differed from earlier life stages.

**Upbringing as a moral legacy and intergenerational tension.** Across interviews, many participants reflected on the way they had raised their children as a values-based practice, emphasizing self-reliance, responsibility, and participation in household tasks. Participants frequently framed this style not only as a necessity shaped by historical conditions, but as a morally “correct” approach to parenting.

At the same time, interview accounts revealed that some great-grandparents now perceive contemporary child-rearing as overly comfortable, emotionally indulgent, and lacking structure. This divergence in parenting expectations was often narrated as generating feelings of emotional distance and relational exclusion in later life. Marie reflects on her past parenting practices with a sense of pride in having instilled responsibility and independence at an early age:

*“They had to learn to dress themselves, do everything on their own… at ten I taught them this, by twelve I taught them how to sort laundry, how to wash, because we did not have an automatic machine… just an ordinary washer… I led them to tidy up… made them wash floors, dishes… I taught them everything.” … “Kateřina still reproaches me for it… that they could have been playing outside like other kids, but they always had to do something… but I just taught them to help me.” (Marie, 76)*

Marie described the differences between her own parenting and her daughter’s views. She expresses pride in having taught her children responsibility and independence early on, yet also acknowledges her daughter’s retrospective reproach—that they were given too many responsibilities and not enough time to play. This tension may reflect what Brannen (2006) calls negotiated intergenerational transmission, in which values and caregiving ideals are revisited and at times contested. While Marie’s account reflects explicit negotiation over parenting values, other narratives illustrate how intergenerational differences in child-rearing expectations may translate into unequal relational access in later life. In such cases, great-grandparents experience not open conflict but gradual relational exclusion. In a similar vein, Eliška reflected in her interview on her limited relational access to her great-grandchild, whom she perceives as emotionally attached primarily to his grandmother:

*“He’s just granny’s little darling… he doesn’t want anyone else… I take him in the stroller, and I can’t even lean toward him… he just doesn’t want anyone… he won’t be with me because he’s not used to being around me. They don’t bring him to visit me at all.”* (Eliška, 80)

This testimony illustrates how intergenerational arrangements around caregiving and access—shaped by earlier and current parenting norms—may limit great-grandparents’ opportunities to establish a meaningful bond. Eliška reported infrequent visits and said the child preferred the grandmother, which limited her opportunity to build a close relationship.

**Emotional rituals as symbolic care.** Interview accounts suggest that in the great-grandparenting phase, everyday caregiving often shifted to small, repetitive, and symbolically meaningful gestures—such as greetings, restraint, shared presence, or expressions of care—that functioned as emotional rituals maintaining relational continuity even in the absence of active caregiving. These small acts helped maintain emotional closeness and a sense of belonging even without daily contact.

*“No, not anymore. They raise them how they raise them. When they complain, I just say: ‘Shh, be glad you have a mom.’”* (Růžena, 79)

Růžena described herself as a supportive presence rather than an authority figure. Her restraint from intervening and her emphasis on emotional presence can be read as a relational ritual—an ongoing moral practice through which she affirms belonging while respecting generational boundaries. Daniela adds a deeper existential dimension to this symbolic care:

*“I’m alone. So, I just tell myself, I’m going to bed, let me wake up one more time, just to live a little more among them. That’s my thinking. But sometimes when I go to bed, and I’ve had enough, I say to myself: maybe I won’t wake up (laughs).”* (Daniela, 82)

In this sense, Daniela’s repeated orientation toward ‘waking up among them’ functions as an emotional ritual, through which family remains a symbolic anchor giving meaning to time, endurance, and continued existence. Emotional proximity and recognition remain vital even when active caregiving roles have faded.

**Space and embodied presence as anchors of continuity.** Participants described how physical presence in shared spaces and bodily interaction became essential tools of familial bonding. When meetings take place in locations with deep family meaning—such as a family home—past, present, and future are symbolically linked. In this context, family is made not through formal roles or words, but through bodily proximity, shared time, and repeated joint activities.

*“When she sees me, she runs over. I always bring her some treats, and yes, yeah, yeah—she starts bringing out toys, not so much now, but when she was younger… she’d pile them all up. They live there now, they built their house where the old one was, tore it down and built their own. A nice house they have built.”* (Petr, 84)

Participants emphasized that being together in person—sharing space, meals, and conversation—helped them feel part of the family even when their everyday involvement was limited.

### Section III: Critical perspective: discursive marginalization of great-grandparenthood in later life

Respondents described great-grandparenthood as a stage of life that is rarely talked about or clearly defined. Unlike earlier family roles, it is not associated with specific expectations or duties. Some participants viewed this as a kind of freedom—they felt they could “just enjoy it”—while others spoke about uncertainty or feeling overlooked. This ambiguity between autonomy and exclusion was a recurring theme in their accounts.

**Namelessness and role uncertainty.** Respondents described the role of a great-grandparent as unclear and rarely discussed within the family. Many said there were no specific expectations or norms attached to it. Instead, they continued to use familiar family terms, most often “grandfather” or “grandmother,” even when referring to relationships with great-grandchildren.

*“I never thought I’d live to see this day. And it happened. […] I didn’t expect it. There were eight boys in my village from my birth year, and none of them is alive now. […] I was glad I made it, but I didn’t know what it meant. I’m a grandfather, that’s what they call me.”* (Oldřich, 78)

This and similar statements show that participants rarely used the word “great-grandparent” and tended to rely on older, established generational identities that felt more familiar and meaningful to them. Some participants said they did not identify with the term “great-grandparent” and preferred to think of themselves simply as grandparents. They often described this stage as relaxed and without obligations.

*“I don’t really feel like a great-grandmother. When someone calls me that, I cringe […] I don’t have to do anything anymore, only if I want to. Parents raise kids, grandparents spoil them. And me? I just enjoy it. […] The girls are affectionate, but I just cuddle them and enjoy the moment. When someone yells at them, I can’t take it. The little one runs to me—I’m her safe haven.”* (Libuše, 86)

Some participants expressed ambivalence about the label “great-grandmother.” They described it as emotionally distant or unnecessary, preferring to identify simply as grandparents. Several said they no longer felt responsible for caregiving but wanted to stay emotionally present in the family. One participant explained that this stage meant freedom from obligations—“I don’t have to do anything anymore, only if I want to”—while still enjoying contact and affection from younger relatives. These accounts show that great-grandparenthood was often experienced as a relaxed, voluntary role rather than an active or clearly defined family position.

**Embodied aging and withdrawal from care.** Another recurring theme was embodied aging as a reason for stepping back from caregiving. Respondents often described their physical condition as a “natural” reason for reduced involvement in family care.

*“That little one… I just spoiled him a bit. I cannot do activities anymore, I can’t run. I just show him pictures or talk to him. But to actually do something with him? I cannot. He would wear me out… I just sit. He is so energetic… I can’t keep up. I’m just happy I can still make it home.”* (Božena, 91)

The respondent links her limited caregiving role directly to her physical abilities. She repeatedly emphasizes that she can no longer “run,” that the child would “wear her out,” and that she “just sits.” Similar statements appeared across other interviews, where participants described tiredness, loss of energy, or the need to “slow down” as natural signs of aging. These accounts suggest that physical condition often defined how participants perceived their capacity to participate in family life.

**Diminished voice and loss of cultural transmission.** A recurring theme across interviews was the tension between emotional closeness and limited influence. Many respondents said they were loved and respected, but no longer involved in decision-making.

*“When I come in, he runs to me— ‘Granny, Granny, good morning!’ That’s joy. But otherwise—I don’t give advice anymore. They do it their own way, different methods, different times. I just watch.”* (Libuše, 86)

This statement shows how respondents value emotional recognition while withdrawing from advice-giving or direct involvement. Several participants described similar experiences when talking about family traditions or religion.

*“I go to church regularly—on Mondays, Wednesdays, and Fridays—since I was 45. I said I’d do it once I retired, and I kept that promise. But no one else in the family goes. I don’t persuade anyone; it’s up to them… I’d like my great-grandson to receive communion one day since he’s been baptized, but it’s up to his parents. Petra (granddaughter)tells me that if he doesn’t want to go to church, he won’t. So, I don’t interfere. I just say—it’s Lent, it’s Ash Wednesday—but whether they observe it, I don’t know.*

*I told Ivana (granddaughter), ‘…you don’t qualify for confirmation if you haven’t been to confession since your first communion, and you don’t go to church.’ Then the priest even mentioned it in a sermon—that some want the sacraments but are never seen at church. I do things my way. The kids just do things their own way. I just hope they might take something from it one day.”* (Anna , 87)

These accounts suggest that great-grandparents often maintain strong personal beliefs and values but rarely expect them to be shared by younger generations. Their participation in family life is mainly emotional and symbolic, expressed through presence and affection rather than advice or authority.

## Discussion and conclusions

This study demonstrates that great-grandparenthood in later life is rarely actively performed, culturally named, or symbolically recognized. Although the biological presence of a fourth generation is becoming increasingly common, respondents seldom framed it as a meaningful role in their timelines or narratives. The analysis revealed that the mere existence of a great-grandchild does not constitute a great-grandparent identity. Rather, this role requires active relational engagement, cultural recognition, and normative framing—what Morgan (2011) describes as the “doing” of family. The findings suggest that great-grandparenthood is often experienced as a liminal identity—present yet undefined, relationally situated yet narratively absent.

From a life course perspective, roles are enacted through transitions and intergenerational nodes. However, the transition into great-grandparenthood was rarely recognized as a turning point. This absence raises theoretical questions about how late-life roles are constructed—silenced—through relational processes and cultural scripts. Critical gerontology helps illuminate how bodily aging, combined with internalized discourses of “appropriate” late-life behavior, can produce symbolic marginality (Katz, 1996; Gilleard & Higgs, 2010). Respondents’ references to bodily frailty as a “natural” reason for retreat reflect this internalized disciplining of aging. Physical limitation was not only experienced but narrated as an expected and acceptable justification for withdrawal from active care, showing how gendered and age-based norms shape the way great-grandparents negotiate their caregiving role. In Katz’s (1996) terms, aging becomes a moral and discursive project—where bodily weakness legitimizes passivity and reaffirms social norms about what is “appropriate” in late life. This aligns with Gilleard and Higgs’s (2010) notion of the “fourth age,” in which older adults are symbolically positioned beyond agency, marked by cultural silence rather than relational engagement. Aging thus appears not only as a physiological trajectory but also as a socially disciplined identity space in which certain forms of relational agency become constrained. This condition, conceptualized here as relational invisibility, underscores how older adults can be structurally excluded from family scripts despite their biological connectedness.

Gender differences in the timelines underscore the persistence of care and productivity norms. Women articulated their biographies through caregiving and emotional labor, while men emphasized occupational histories and spatial rootedness. This finding resonates with previous research highlighting gendered trajectories of caregiving and family continuity, in which women’s biographies are structured around service, responsibility, and moral commitment to family life (Pacheco Barzallo et al., 2024; Gibson & Houser, 2007). Yet even within these gendered trajectories, great-grandparenthood failed to emerge as a meaningful category. Its symbolic absence persisted regardless of narrative framing, suggesting that the role lacks sufficient cultural anchoring across the board.

These findings invite a broader reflection on the normative and institutional vacuum surrounding the fourth generation. Unlike parenthood or grandparenthood—roles supported by rituals, media representations, and often policy frameworks—great-grandparenthood remains culturally and politically unanchored. This raises critical questions: What might it mean to institutionalize this role? Could family policy or caregiving programs integrate great-grandparents as symbolic or supportive figures, especially in aging societies? Or would such attempts risk turning an ambivalently open role into a prescriptive and potentially burdensome identity? Rather than answering these questions definitively, this study suggests the need to politicize the seemingly private domain of late-life family roles. Great-grandparenthood offers a unique lens through which to reimagine aging, intergenerationally, and identity in the 21st century. Rather than viewing old age as a phase of symbolic retreat, we might recognize it as a site of moral presence, cultural continuity, and relational anchoring. By legitimizing the symbolic and emotional contributions of very old adults, societies can construct more inclusive narratives of family belonging across generations.

A further dimension concerns the symbolic silencing of great-grandparents within intergenerational communication. While they are emotionally appreciated, their authority and voice are often muted. This dynamic resonates with what has been described as epistemic injustice (Fricker, 2009), insofar as age and generational position may undermine the perceived credibility of great-grandparents’ knowledge and perspectives. As Bourdieu (1991) suggests, symbolic power structures define who is recognized as a legitimate speaker within the family. The result is a subtle exclusion: great-grandparents’ stories and values, though still performed, are seldom granted interpretive weight. Religion, once a shared intergenerational language, becomes a private practice of resilience (Wild et al., 2011), maintained without expectation of transmission. In this sense, great-grandparenthood becomes not only relationally invisible but also narratively muted—a form of “doing family” (Morgan, 2011) enacted more through presence than through voice. The ambivalence expressed by participants who resisted identifying as “great-grandparents” reflects how rigid cultural age norms shape late-life subjectivity and constrain the ways older adults can enact family roles. This subtle resistance exemplifies what van Dyk (2014) calls cultural ageism—the normative framework that associates later life with decline and diminished social value. The label “great-grandmother” often evokes connotations of fragility and loss of vitality, which may discourage older women from embracing it. The absence of a clear cultural script for this role thus becomes paradoxical: it provides a sense of freedom (“I don’t have to do anything”) while simultaneously erasing normative recognition, legitimacy, and protection. As Lamb (2014) suggests, this creates a form of ambiguous autonomy, in which independence from family obligations may be experienced as empowerment yet also reinforces social marginality. Participants’ insistence that they “don’t have to do anything anymore” illustrates how ideals of active aging can coexist with quiet withdrawal. In this sense, cultural ageism (van Dyk, 2014) operates not through explicit exclusion but through the normalization of self-limiting narratives that frame disengagement as appropriate or even desirable.

Ultimately, great-grandparenthood emerges not from biology alone but from processes of recognition, narration, and relational engagement. Its invisibility reflects broader cultural scripts that fail to name and validate this role. As such, the concept of relational invisibility may extend beyond great-grandparenthood, offering a critical tool for analyzing other late-life roles that remain unspoken, unsupported, and under-theorized. Taken together, these dimensions—embodiment, symbolic silencing, and ambiguous autonomy—illustrate the paradoxical position of great-grandparenthood within late-life family structures. It is simultaneously a site of connection and disconnection, of presence without recognition. The role’s cultural invisibility is therefore not accidental but produced through intersecting age norms, discursive hierarchies, and the moralization of independence in later life.

Future research could explore how younger generations perceive great-grandparents, how this role is represented in media and education, or how different cultural contexts may enable or constrain its performance. In this way, rethinking great-grandparenthood opens the door not only to a deeper understanding of family life and aging, but also to a broader critique of whose contributions are seen, named, and valued within families—and within societies—over time.

### Methodological reflections and limitations

This study was designed to offer a deep, context-sensitive understanding of how great-grandparenthood is perceived and enacted in later life. The research sample consisted of ten participants selected based on their status as great-grandparents and their ability to reflect on life events and intergenerational relationships. Rather than aiming for statistical representativeness, the study prioritized narrative depth, first-person meaning-making, and the exploration of life course trajectories. All participants were cognitively capable and able to participate in a qualitative interview, with particular attention paid to ethical considerations related to age, vulnerability, and informed consent.

The focus on great-grandparents’ own perspectives enabled a rich account of how this role is experienced from within. However, this single-generational lens also represents a limitation, as it excludes the perspectives of other family members through which intergenerational roles are co-constructed. Similarly, the small, purposively selected sample limits the generalizability of findings; the insights presented here should therefore be interpreted as contextually grounded rather than representative.

The use of visual timelines proved particularly effective in eliciting biographical structure and relational rhythm. At the same time, this approach required a certain degree of temporal orientation, memory recall, and visual imagination, which may have shaped the narratives produced. This methodological choice may have favored participants who were more reflective or verbally expressive.

The co-constructed nature of the interviews must be acknowledged. Meanings and narratives emerged through interaction between researcher and participant and should be understood as dialogically produced rather than fixed or solely individual accounts. As a younger researcher, it was recognized that generational position may have influenced participants’ openness and interpretation, requiring ongoing reflexivity throughout the research process. These reflections highlight both the depth and the limits of qualitative inquiry: the aim was not to generalize but to illuminate the lived, relational, and narrative dimensions of great-grandparenthood within a specific cultural and historical context.

### Implications for theory, family practice, and future research

This study contributes to gerontological and family scholarship by further specifying how great-grandparenthood is relationally enacted rather than institutionally defined. By combining life course theory, the concept of doing family, and narrative gerontology, the findings extend existing discussions of intergenerational roles beyond caregiving and dependency, highlighting symbolic presence, emotional rituals, and relational recognition as key dimensions of very late-life family engagement.

The findings also have implications for family and caregiving practices. They suggest that great-grandparents’ involvement often occurs outside formal caregiving arrangements and may therefore remain unnoticed or undervalued within family decision-making. Recognizing symbolic and relational forms of participation may help families and practitioners better acknowledge older adults’ contributions to intergenerational continuity, emotional support, and moral orientation, even in the absence of active care.

Finally, this study points to several directions for future research. Further studies could incorporate intergenerational perspectives by including parents, grandparents, and great-grandchildren, allowing for a more relational analysis of role negotiation and recognition. Comparative research across cultural and institutional contexts could also illuminate how different family norms and welfare regimes shape the visibility and meaning of great-grandparenthood. Longitudinal and mixed-method approaches would further enhance understanding of how late-life family roles evolve over time.

## Data Availability

The data supporting the findings of this study are not publicly available due to ethical and privacy considerations. Analytical materials are available upon reasonable request.
